# From Cocoa to Chocolate: The Impact of Processing on *In Vitro* Antioxidant Activity and the Effects of Chocolate on Antioxidant Markers *In Vivo*

**DOI:** 10.3389/fimmu.2017.01207

**Published:** 2017-09-29

**Authors:** Carla D. Di Mattia, Giampiero Sacchetti, Dino Mastrocola, Mauro Serafini

**Affiliations:** ^1^Faculty of Biosciences and Technologies for Agriculture, Food and Environment, University of Teramo, Teramo, Italy

**Keywords:** cocoa, chocolate, processing, polyphenols, antioxidant activity, chronic intervention studies

## Abstract

Chocolate is a product processed from cocoa rich in flavonoids, antioxidant compounds, and bioactive ingredients that have been associated with both its healthy and sensory properties. Chocolate production consists of a multistep process which, starting from cocoa beans, involves fermentation, drying, roasting, nib grinding and refining, conching, and tempering. During cocoa processing, the naturally occurring antioxidants (flavonoids) are lost, while others, such as Maillard reaction products, are formed. The final content of antioxidant compounds and the antioxidant activity of chocolate is a function of several variables, some related to the raw material and others related to processing and formulation. The aim of this mini-review is to revise the literature on the impact of full processing on the *in vitro* antioxidant activity of chocolate, providing a critical analysis of the implications of processing on the evaluation of the antioxidant effect of chocolate in *in vivo* studies in humans.

## Introduction

Chocolate, thanks to its unique structure and flavor, is a food usually consumed for pleasure that has been recently reconsidered as a source of healthy compounds. Chocolate is rich in polyphenols such as flavanols, which possess antioxidant and anti-inflammatory properties and have a protective effect against degenerative diseases (1–6). Procyanidin and flavanol polymers also contribute to chocolate taste by affecting bitterness and astringency (7, 8). The polyphenol content of chocolate depends on many factors, some related to the raw material, and others related to processing (9, 10).

The majority of published reviews aim at analyzing the impact of processing on the polyphenol content of cocoa more than on its functional properties, focusing only on selected processing steps deemed to have a major impact on phenolic content, and, sometimes, without a specific discussion of all the single steps (9–11).

This mini-review aims at revising the literature on the impact of full processing on the *in vitro* antioxidant properties of chocolate providing a critical analysis of the implication of processing on the antioxidant effect of chocolate in *in vivo* studies in humans.

## Chocolate Processing in Brief

Chocolate-making consists of a multistep process. At harvest, cocoa fruit contains about 30–40 seeds covered by a mucilaginous pulp removed by yeast and bacteria during fermentation, which is a key step for the development of the chocolate flavor, since it produces aroma precursors. After fermentation, a drying step is required to reduce the water content to 5–7%; this ensures product stability before further processing. Dried cocoa beans or nibs (i.e., beans without the outer shell) are then roasted to further develop the chocolate flavor. The next step in cocoa processing involves nib grinding to convert the solid nibs into a liquid paste (liquor).

For the production of dark chocolate, the basic ingredients are cocoa liquor, sugar, cocoa butter, and emulsifiers. Milk and other ingredients may be added, mixed and then refined to reduce the particle sizes of solids. After refining, the conching operation, which consists of the agitation of the chocolate mass at high temperatures, and finally tempering, which consists in a heating, cooling and mixing process, are required for the development of the final texture and flavor.

## Phenolic Antioxidants in Chocolate

Polyphenols are the main class of antioxidants in unfermented cocoa beans, and they account for approximately 2% w/w (12). Cocoa contains several classes of phenolic compounds among which, flavanols (37%), proanthocyanidins (58%), and anthocyanins (4%) (11).

Flavanols, and, in particular, flavan-3-ols, are the most studied compounds in cocoa. The main flavan-3-ols, are (−)-epicatechin and (+)-catechin, which have an antioxidant activity of 2.4–2.9 trolox equivalents (TE) using the 2,2′-azino-bis(3-ethylbenzothiazoline-6-sulfonic acid) (ABTS) assay and 2.2 TE using the ferric reducing antioxidant power (FRAP) assay, but they can be epimerized into (+)-epicatechin and (−)-catechin during processing into chocolate (5, 13).

Flavan-3-ols may group together to form dimeric, oligomeric, or polymeric combinations of units that are denominated proanthocyanidins, among which we can include procyanidins (oligomers of epicatechin). Oligomeric and polymeric proanthocyanidins are present in raw beans but could further polymerize during processing (14–16). The procyanidin dimers (B1, B2, B3, and B5) and trimer C1, as well as oligomers, up to decamers, have been reported in cocoa and chocolate (12, 17–19). The average antioxidant activity of procyanidin dimers is about 6.5, and that of trimers is 7–8 TE using the ABTS assay. Monomers, dimers, and trimers account for almost 33% of the antioxidant activity of cocoa. The antioxidant activity of procyanidin polymers seems to increase depending on the degree of polymerization even though polymerization decreases the concentration of polyphenols; the relative contribution of decamers to the total antioxidant activity is low (14).

Esters of catechins, such as gallocatechins and epigallocatechins, can be found in raw beans (20) but could also be formed during processing, in particular, during roasting (16), whereas esters of epigallocatechins, such as epigallocatechingallate, have only been reported in chocolate (21).

Anthocyanins that have been reported in fresh beans (22) are degraded during fermentation due to hydrolysis and further polymerization in condensed tannins (20).

Minor phenolic compounds are also present (i.e., flavonols, phenolic acids, simple phenols and isocoumarins, stilbenes, and their glucosides), but their content is low and their contribution to total antioxidant activity is limited.

Apart from polyphenols, chocolate contains other process-derived antioxidants such as Maillard reaction products (MRPs) that form during high temperature processing, among which drying, roasting, and conching.

## Effect of Cocoa Processing on Antioxidant Activity

The evaluation of the antioxidant (i.e., phenolics) content and activity much depends on the extraction solvent and procedure (9), which is not standardized throughout literature on cocoa, so data are difficult to compare. In the colorimetric assays of the total phenolic content (TPC), discrepancies may arise due to the phenolic compounds used as reference for the standard curve as well as to the presence of reducing compounds, interfering with the assay. Regarding antioxidant activity, comparison of results could be problematic due to the large number of heterogeneous tests used. The most common assays [ABTS, 2,2-diphenyl-1-picrylhydrazyl (DPPH), oxygen radical antioxidant capacity, total radical-trapping antioxidant parameter (TRAP), and FRAP] are based on different reaction mechanisms (single electron transfer, hydrogen atom transfer, or mixed mechanisms) and could give discordant results depending on the most abundant antioxidant molecules in the system and their interactions.

### Cocoa Beans

Cocoa beans are the seeds of the tropical *Theobroma cacao* L. tree. There are four types of cocoa: Forastero, which comprises 95% of the world production of cocoa and is the most widely used; Criollo, which is rarely grown because of disease susceptibility; Trinitario, which is a more disease-resistant hybrid of Criollo and Forastero; and Nacional, which is grown only in Ecuador (20, 23). The concentration of phenolic compounds in cocoa beans is highly variable and depends primarily on genetics, and then on many other factors such as geographical regions of cultivation, agronomical practices and climatic conditions (20).

Generally, Criollo cocoa beans have a lower phenolic content compared to the Forastero variety (10). Unfortunately, few studies on the phenolic content and antioxidant properties of unfermented beans are available and most results refer to beans that have undergone fermentation, drying or both these processes. When unfermented beans are considered, the total phenolic content results in a range between 67 and 149 mg/g (24) or 120 and 180 mg/g (25). Large differences in the content of total polyphenols and individual phenolic compounds in unfermented ripe seeds of Forastero, Trinitario, and Criollo cocoa of six different origins were reported (22). Antioxidant activities of 709 ± 17 µM and 240–490 mmol TE/g were reported when the DPPH test was used (26, 27); however, the tests differed as regards the experimental conditions adopted. Values of 1.29–2.29 mmol TE/g and 600–800 mmol TE/g_dw_ were found with the ABTS method (14, 27) while reducing activities in the range 713–930 mmol Fe^2+^/g_dw_ were obtained when using the FRAP method (14).

### Fermentation

Fermentation of the pulp surrounding the beans represents the first important step for the development of chocolate flavor and taste since it produces aroma precursors. During fermentation, which can last from 5 to 10 days, the combination of endogenous and microbial enzymatic activities, along with the rise of temperature to about 50°C, and the diffusion of metabolites into and out of the cotyledons, allow polyphenols to polymerize and react with other compounds to form complexes. Fermentation is thus considered responsible for the decrease of the flavan-3-ol content, (−)-epicatechin in particular.

The level of polyphenol reduction is proportionate to the degree of fermentation (25, 28–30). Significant differences can be detected in the TPC content after fermentation as determined by the Folin–Ciocalteu’s reagent: a range between 120–140 mg/g was found by Di Mattia et al. (14); a similar range (90–120 mg/g) was reported by Niemenak et al. (24) and Afoakwa (20). Higher levels (220 mg/g) were detected by Ryan et al. (31) while lower contents were determined by do Carmo Brito et al. (32). The antioxidant activity, as determined by the ABTS, DPPH, and FRAP methods, generally followed the same fate of the phenolic content, with reduction levels of 20–40% (14, 32). In the work by Suazo et al. (26), a reduction of about 80% was determined in the DPPH values while an increase in the total antioxidant capacity (+50–160%), evaluated using DPPH and ABTS methods, was observed in cocoa varieties after spontaneous fermentation (27).

### Drying

The aim of cocoa drying is to remove water so as to reach moisture content below 7% and is usually carried out by sun heating in static conditions but heating dryers are also used.

Sun drying reduces the polyphenol content to different extents: Camu et al. (29) reported a reduction from 77 to 44%, Di Mattia et al. (14), a 72% reduction, Hii et al. (11), a 30% reduction, and finally, de Brito et al. (28), a 26% reduction. The reduction of polyphenols depends on climatic conditions (29), and reduction levels ranging from 77 to 44% were reported for the same cocoa sample dried in different seasons.

Sun drying not only affects the polyphenol content but also the antioxidant activity of cocoa beans, and a reduction of about 70% of TPC and 80% in flavan-3-ols was shown to determine a decrease of 70 ± 5% in antioxidant activity depending on the method used (14).

Experimental data on air drying are scarce; an industrial process carried out on a batch of 1,600 kg of cocoa beans for 11 days at a temperature of 60°C, decreased the content of TPC (52%) and flavan-3-ols (66%) inducing a 60 ± 5% decrease of antioxidant activity, depending on the assay (14). Hot air drying of cocoa beans has also been studied in laboratory scale conditions (11, 33–36), and the mean reduction of total polyphenols was about 45%, but this could dramatically change depending on process conditions.

### Roasting

Roasting determines the formation of the characteristic color, aroma, taste, and texture of roasted cocoa beans (37). Roasting temperatures of 120–150°C and times of 5–120 min are used (37, 38), and under these conditions, a decrease of flavanols and TPC has been observed.

During roasting, monomeric flavanols are reduced from 0 to 95% depending on the cultivar and the roasting temperature (16, 18, 19). High roasting temperatures improve the rate of polyphenol degradation, but in some cases a lower degradation was observed at high temperatures due to reduced processing times (16). Roasting temperature being equal, polyphenol degradation could be reduced by about 20% by adopting “high” relative humidity (5%) roasting conditions (18).

Roasting generally depletes the antioxidant activity of cocoa. Arlorio et al. (39) reported a decrease between 37 and 48% after pre-roasting at 100°C and roasting different varieties of cocoa at 130°C. Hu et al. (40) reported a decrease of antioxidant activity between 44 and 50% during roasting at high temperature (190°C) for short times (15 min) regardless of the assay used to test it. Ioannone et al. (16) observed a decrease of antioxidant activity during the first part of the roasting process and an increase during roasting time due to the formation of MRPs (16, 41). They reported a FRAP decrease of 51 and 45% at 125 and 145°C, respectively, as well as a TRAP increase of 7% at 125°C and a TRAP decrease of 20% at 145°C at the end of roasting. Dramatic differences between FRAP and TRAP values could be explained by considering MRP formation during roasting (41) since MRPs show a high chain-breaking activity despite their low reducing potential (42). A low roasting temperature (125°C) led to higher TRAP values but lower FRAP values than a high roasting temperature (145°C).

### Conching

Conching is a unit operation based on the agitation of chocolate mass at high temperatures (above 50°C); it is an essential step for the development of proper viscosity and the attainment of final texture and flavor (23, 43). Different time/temperature combinations are selected according to the final product to be manufactured. In dark chocolates, temperatures ranging from 70 to 90°C can be used; variations in conching time and temperature combinations modify chocolate texture and flavor (44–46). Little attention has been paid to conching and its effect on polyphenol content and antioxidant properties. However, the conching process does not impair the phenolic content and pattern, as well as antioxidant activity since small yet not significant variations (3%) were found, regardless of the time/temperature combination applied (47–49). The same results were reported by Di Mattia et al. (15) for the TPC; however, authors reported a significant increase of trolox equivalent antioxidant capacity (+16% on average) after conching.

### Complete Process

The content and antiradical activity of cocoa beans, nibs, cocoa mass, and finished dark chocolate obtained from fermented beans from different geographical origins have been studied (50). Generally a progressive decrease of the phenolic content was observed upon processing, with roasting playing a major role. Nonetheless, the most significant losses in both phenolic content and antioxidant activity emerged in the final steps of processing, and in particular between the conched and non-tempered chocolate and the dark chocolate. The authors remarked that the results were ascribable to a dilution and even to an antagonistic effect produced by the addition of other ingredients. However, it is not clear if the authors considered the recovery of phenolic compounds on the basis of the amount used in the recipe (40% of cocoa mass).

**Figure 1:**
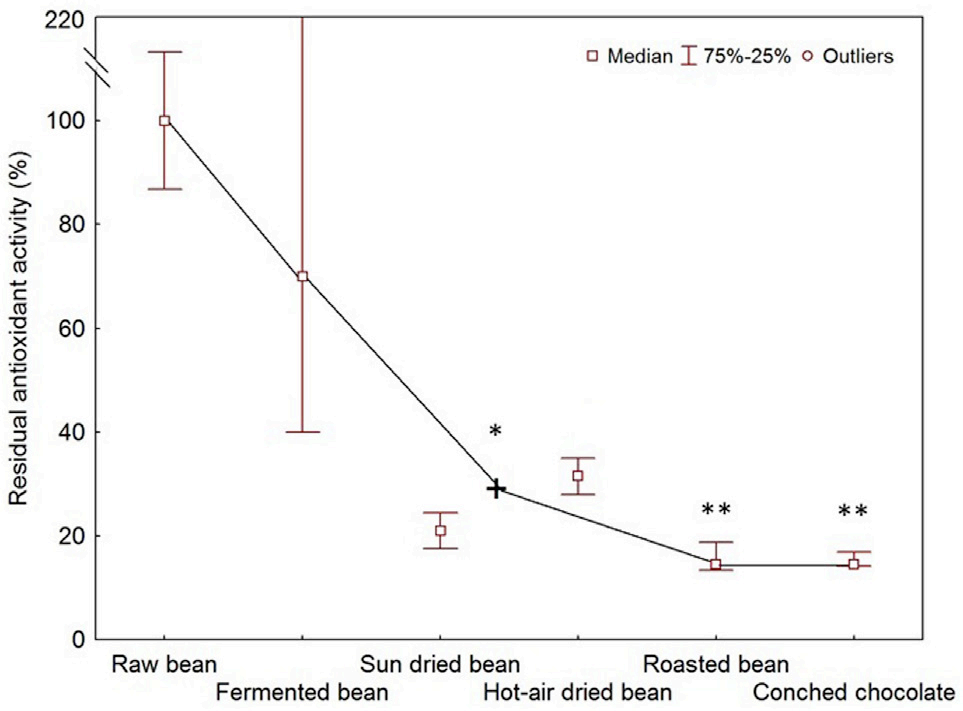
Residual antioxidant activity of cocoa processed products after each processing step. *Mean of sun drying and hot air drying data; **data calculated on the mean of sun drying and hot air drying data. The top of the error bar of the second point on the *x*-axis overlaps with the figure frame.

Despite few attempts, the concurrent evaluation of the changes of polyphenol content and antioxidant activity upon all the processing steps is actually lacking and further investigations are needed. A general trend of the variation of antioxidant activity during processing is shown in Figure 1, obtained by taking into account the losses reported in works where single manufacturing steps were considered.

## Antioxidant Effect of Chocolate *In Vivo*

As far as chronic intervention studies in humans are concerned, there are no published studies that consider the effect of processing on the antioxidant properties of chocolate. This is a big gap in literature that deeply impairs the massive amount of work performed on chocolate processing optimization.

Literature data from 10 human chronic intervention studies investigating the effect of chocolate intake on plasma and urinary levels of markers of antioxidant function, isoprostanes, and non-enzymatic antioxidant capacity (NEAC) were reviewed, and the results are presented on Table 1, where type of chocolate, number of intervention days, number of subjects, dose/day, effect on isoprostanes, effect on NEAC, and effect on polyphenols were described. Plasma/serum/urine isoprostanes, plasma NEAC, and polyphenols were assessed in nine, six, and seven studies, respectively.

**Table 1:** Chronic intervention studies in humans providing cocoa-based products: effect on F_2_-IsoP, NEAC,^a^ and PP.^a^

Food	Days	Subjects	Dose/day	F_2_-IsoP	NEAC^a^	PP^a^	Reference
Flavonoid-rich dark chocolate	14	11	46 g	↔ Plasma	↔	↑ EC	(2)
Cocoa tablets	28	13	6 Tablets	↔ Plasma	↔	↑ EC, C	(51)
Dark chocolate and cocoa powder drink	42	25	36.90 g of dark chocolate and 30.95 g of cocoa powder drink	↔ Urine	↔	↔ Total phenols	(52)
Dark chocolate	21	15	75 g	↔ Plasma	↔		(53)
Polyphenols-rich dark chocolate	21	15	75 g	↔ Plasma	↔		(53)
Polyphenols-rich dark chocolate	126	22 with prehypertension or stage 1 hypertension	6.3 g	↔ Plasma		↔ EC, C, procyanidin B2, procyanidin B2 gallate	(54)
PP-rich milk chocolate	14	28	105 g		↔	↔ C, EC	(55)
Flavonoid-rich dark chocolate	14	20	45 g	↔ Serum			(56)
Dark chocolate	14	19 NASH 1	40 g	↓ Serum		↑ ECMet, TP	(57)
Milk chocolate	14	19 NASH 1	40 g	↔ Serum		ECMet,^b^ ↔ TP	(57)

*^a^Plasma and/or serum measurements*.

*↑, increase; ↔, no change; ↓, decrease; F_2_-IsoP, F_2_-isoprostanes; NEAC, non-enzymatic antioxidant capacity; PP, polyphenols; NASH, non-alcoholic steatohepatitis; EC, epicatechin; C, catechin; ECMet, epicatechin-3O-methylether*.

*^b^Discrepancy between table and text. Modified from Petrosino and Serafini (58)*.

On the basis of existing data, only one study showed an effect of chocolate on markers of antioxidant functions in humans. An increase in plasma polyphenol levels, namely, epicatechin, catechin, epicatechin-3*O*-methylether, and total phenolics, following a cocoa-based product supplementation period was detected in three studies out of seven. Increases were not correlated to any changes in markers of antioxidant function except for Loffredo et al. (57).

Although, from this analysis, it could be inferred that antioxidant networks do not respond very well to dietary supplementation with chocolate, some considerations are required. First of all, we need to consider the high heterogeneity of the reviewed studies, involving not only very different chocolate sources and doses of supplementation but also different size power, type of subjects, and duration of the supplementation; all variables that might affect the outcome of the trial.

It seems that all the different formulations that were used in the studies, such as tablets and chocolate drinks, failed to display any significant effect. Moreover, in agreement with previous evidences *in vivo* (1), milk chocolate does not produce any significant antioxidant effect in humans, and it has been utilized as control (57) in the only study where an effect was detected with dark chocolate.

The outcome of a study may also depend on the kind of subjects involved, namely, on their health condition. As previously stated, elevated levels of isoprostanes have been reported in individuals with diseases, or related risk factors, in which oxidative stress is involved; these subjects are supposed to have a higher requirement of antioxidants and, thus, to better respond to dietary intervention. In this respect, it is interesting to highlight that the only study where chocolate displayed an antioxidant effect in humans was conducted on subjects with non-alcoholic steatohepatitis diseases characterized by a non-physiological condition of oxidative stress. When oxidative stress is ongoing, endogenous antioxidants are not able to inhibit the production of free radicals efficiently; therefore, the contribution of exogenous antioxidants in diets may be crucial to support the endogenous redox system providing a clear effect on antioxidant status markers in humans (59–61). This aspect might explain the lack of effect observed for chocolate products, since all the studies, except the one where chocolate was effective, were conducted on healthy subjects characterized by a physiological equilibrium of free radicals and antioxidants. A systematic review (62) and a meta-analysis (63) support this hypothesis by showing that plant food, as well as chocolate supplementation, displays a better efficiency on antioxidant defense markers when the trials are conducted on subjects with oxidative stress-related risk factors rather than on healthy subjects. Moreover, in a large clinical trial on subjects characterized by cardiovascular disease risk factors, the PREDIMED study, it was shown that the efficiency of the supplementation of Mediterranean diet with antioxidant rich foods for 1 year was correlated with the baseline levels of antioxidant defenses (64). Subjects starting from lower levels of plasma NEAC showed a higher increase in NEAC compared to subjects starting from higher baseline levels of antioxidants, highlighting the importance of the redox “condition” of the subject on the efficiency of antioxidant supplementation.

## Conclusion

Chocolate processing affects the content of total polyphenols as well as the antioxidant activity of chocolate and proper technology could “optimize” polyphenol retention and the *in vitro* antioxidant activity of chocolate. This work highlights the need to provide evidence of chocolate functionality in human beings to identify a proper technological process for chocolate processing. This is a necessary step to suggest to consumers the “optimal” doses of chocolate, which optimizes the functional effect by avoiding potential side effects, such as a high-energy load.

Human trials should be conducted mainly on subjects characterized by oxidative stress conditions, sharing a common requirement for dietary antioxidants, to increase the chance of observing an antioxidant effect *in vivo*.

## Author Contributions

CM, GS, and DM contributed to draft the section related to food chemistry and technology; MS contributed to draft the section related to nutrition; and CM, GS, DM, and MS contributed to data analysis and interpretation.

## Conflict of Interest Statement

The authors declare that the research project was conducted in the absence of any commercial or financial relations that could be construed as a potential conflict of interest.

## Abbreviations

ABTS, 2,2′-azino-bis(3-ethylbenzothiazoline-6-sulfonic acid); DPPH, 2,2-diphenyl-1-picrylhydrazyl; FRAP, ferric reducing antioxidant power; MRPs, Maillard reaction products; TE, trolox equivalents; TEAC, trolox equivalent antioxidant capacity; TPC, total phenolic content; ORAC, oxygen radical antioxidant capacity; TRAP, total radical-trapping antioxidant parameter; NEAC, non-enzymatic antioxidant capacity; NASH, non-alcoholic steatohepatitis; CVD, cardiovascular disease.
